# The NTI-tss device for the therapy of bruxism, temporomandibular disorders, and headache – Where do we stand? A qualitative systematic review of the literature

**DOI:** 10.1186/1472-6831-8-22

**Published:** 2008-07-29

**Authors:** Henrike Stapelmann, Jens C Türp

**Affiliations:** 1 Clinic for Reconstructive Dentistry and Temporomandibular Disorders, Dental School, Hebelstrasse 3, 4056 Basel, Switzerland; 2 Interuniversity College for Health and Development Graz/Castle of Seggau, Austria

## Abstract

**Background:**

The NTI-tss device is an anterior bite stop, which, according to the manufacturer, is indicated for the prevention and treatment of bruxism, temporomandibular disorders (TMDs), tension-type headaches, and migraine. The aim of this systematic review was to appraise the currently available evidence regarding the efficacy and safety of the NTI-tss splint.

**Methods:**

We performed a systematic search in nine electronic databases and in NTI-tss-associated websites (last update: December 31, 2007). The reference lists of all relevant articles were perused. Five levels of scientific quality were distinguished. Reporting quality of articles about randomized controlled trials (RCTs) was evaluated using the Jadad score. To identify adverse events, we searched in the identified publications and in the MAUDE database.

**Results:**

Nine of 68 relevant publications reported about the results of five different RCTs. Two RCTs concentrated on electromyographic (EMG) investigations in patients with TMDs and concomitant bruxism (Baad-Hansen et al 2007, Jadad score: 4) or with bruxism alone (Kavaklı 2006, Jadad score: 2); in both studies, compared to an occlusal stabilization splint the NTI-tss device showed significant reduction of EMG activity. Two RCTs focused exclusively on TMD patients; in one trial (Magnusson et al 2004, Jadad score: 3), a stabilization appliance led to greater improvement than an NTI-tss device, while in the other study (Jokstad et al 2005, Jadad score: 5) no difference was found. In one RCT (Shankland 2002, Jadad score: 1), patients with tension-type headache or migraine responded more favorably to the NTI-tss splint than to a bleaching tray. NTI-tss-induced complications related predominantly to single teeth or to the occlusion.

**Conclusion:**

Evidence from RCTs suggests that the NTI-tss device may be successfully used for the management of bruxism and TMDs. However, to avoid potential unwanted effects, it should be chosen only if certain a patient will be compliant with follow-up appointments. The NTI-tss bite splint may be justified when a reduction of jaw closer muscle activity (e.g., jaw clenching or tooth grinding) is desired, or as an emergency device in patients with acute temporomandibular pain and, possibly, restricted jaw opening.

## Background

In July 1998, the U.S. Food and Drug Administration (FDA) granted approval for the "NTI Clenching Suppression System" (now: "Nociceptive Trigeminal Inhibition Tension Suppression System": NTI-tss). According to the manufacturer, the NTI-tss device is indicated for the prevention and treatment of bruxism, temporomandibular disorders (TMDs), occlusal trauma, tension-type headaches and/or migraine [1].

**Figure 1:**
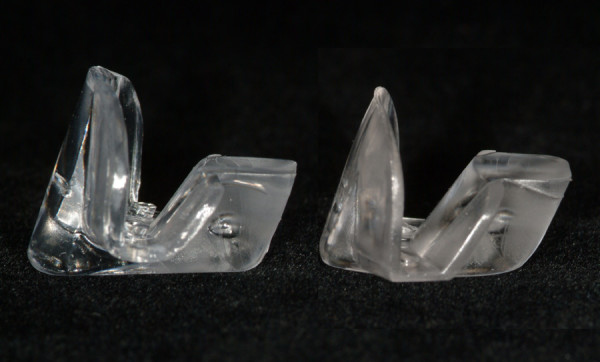
The NTI-tss device, standard type (left) and vertically reduced type (right).

**Figure 2:**
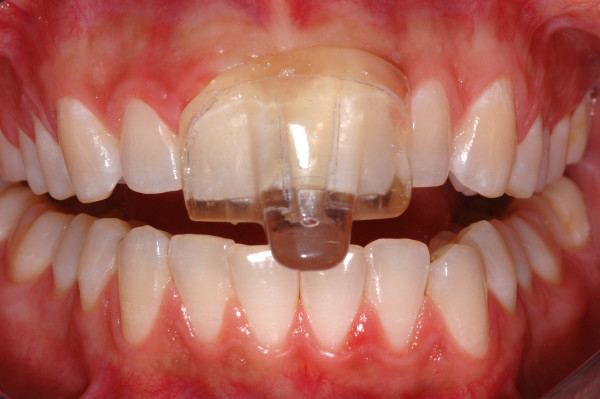
Inserted NTI-tss device.

The NTI-tss device is a small pre-fabricated anterior bite stop (Figure 1) which covers – in its most widely used form – the two maxillary (or mandibular) central incisors (Figure 2). The fit along the teeth is accomplished at the chair side by filling either an autopolymerizing acrylate or a thermoplastic material into the base of the device, which is subsequently adapted along the central incisors, thereby increasing the vertical dimension between the upper and lower jaw. Adjustments along the outer surface of the bite stop are made by the dentist to ensure that at jaw closure and during excursive movements tooth contacts are present only between the intraoral device and the incisal embrasures of the antagonistic teeth. This "miniature anterior bite appliance" [2] is typically worn during the night, although two variations of the bite stop are offered for daytime use [3].

In the lay press, most articles have reported positively about the NTI-tss splint in the therapy of long-lasting headache or facial pain [e.g., [4-6]]. Likewise, excited dental practitioners have published their personal impressions in local dental journals [e.g., [7]] or on the website of the international NTI-tss provider e-motion [3].

Conversely, some renowned clinical researchers [e.g., [8-12]] have tempered over-optimistic expectations by raising doubts on the claimed success and by pointing at the possibility of unwanted side effects, such as aspiration, ingestion, occlusal changes after prolonged unmonitored use, and mobility of anterior teeth. In 2003, Helkimo [8] delivered an expert statement on demand of the Swedish Dental Association and the Swedish National Board of Health and Welfare on the question whether the use of the NTI-tss device "is to be regarded as lege artis and according to science and empirical experience, both as to the treatment of stomatognathic problems as well as migraine." The author came to the conclusion that there is a "total lack of scientific documentation of its therapeutic effects and possible unwanted side-effects" [8]. As far as side effects are concerned, Jokstad et al [10] mentioned that one person in Norway [13] and three individuals in the United States were subjected to medical emergencies due to aspirated NTI-tss devices splints. For the three cases from the U.S., the author referred to the FDA's Manufacturer and User Facility Device Experience Database (MAUDE), which contains voluntary, user facility, distributor, and manufacturer reports of adverse events involving medical devices. Later, Wright and Jundt [12] repeated the contention of four aspirated NTI-tss devices by referring Jokstad et al's article [10].

Considering the controversy that exists within parts of the international dental community regarding the benefits and dangers of the NTI-tss device, it appears to be timely to

(a) systematically review the (dental) literature in order to summarize and appraise the currently available data on the efficacy and/or effectiveness of the NTI-tss device, and

(b) to summarize all identifiable documented cases, in which complications and/or side effects associated with this anterior bite stop have been reported in the dental literature.

## Methods

### Identification of publications about the efficacy and/or effectiveness of the NTI-tss device

To identify a maximum number of relevant publications (including dental congress abstracts), we performed a systematic search in the following electronic databases:

1. The Cochrane Library

2. PubMed

3. TRIP database

4. MEDPILOT.DE

5. BIREME

6. The database of the Deutscher Ärzte-Verlag, which comprises the four German-language dental journals "Deutsche Zahnärztliche Zeitschrift", „Zeitschrift für Zahnärztliche Implantologie“, „Oralprophylaxe & Kinderzahnheilkunde“, and „Zahnärztliche Mitteilungen“

7. The database of the Quintessenz Verlag, which considers the seven German-language dental journals "Die Quintessenz", „Kieferorthopädie“, „Parodontologie“, „Implantologie“, „Endodontologie“, „Quintessenz Team-Journal“ and „Quintessenz Zahntechnik“

8. Google Scholar

9. Web of Science (Cited reference search)

The key words and strategies for the searches in each of the nine databases are shown in Tables 1 to 9. Moreover, were searched NTI-tss-associated websites for relevant articles and references.

**Table 1:** The Cochrane Library.

**#**	**Search terms**	**Hits**	**Relevant hits**	**Cumulative relevant hits**	**Reference**
1	NTI	10	2	2	[9,10]
2	NTI-tss	0			
3	NTI*	Cochrane reviews: 11	0		
		clinical trials: 37	2	2	
		methodological studies: 2	0		
		economic evaluation: 1	0		
4	nociceptive trigeminal inhibition	5	4	5	[16-18]
5	nociceptiv* trigeminal inhibition	6	5	5	
6	anterior deprogrammer	0			

Search strategy and results (search date: December 31, 2007)

**Table 2:** PubMed.

**#**	**Search terms**	**Hits**	**Relevant hits**	**Cumulative relevant hits**	**(New) Relevant references**
1	NTI	195	7	7	[9,10,12,22,60-62]
2	NTI-tss	0			
3	NTI*	355	7	7	
4	nociceptive trigeminal inhibition	107	4	10	[16-18]
5	nociceptiv* trigeminal inhibition	108	5	10	
6	anterior deprogrammer	2	0		
7	"Temporomandibular Joint Disorders"[MeSH] AND NTI	7	7	10	
8	"Temporomandibular Joint Disorders"[MeSH] AND NTI-tss	0			
9	"Temporomandibular Joint Dysfunction Syndrome"[MeSH] AND NTI	0			
10	"Temporomandibular Joint Dysfunction Syndrome"[MeSH] AND NTI-tss	0			
	**Clinical queries; therapy, broad sensitive search:**				
11	(NTI) AND ((clinical [Title/Abstract] AND trial [Title/Abstract]) OR clinical trials [MeSH Terms] OR clinical trial [Publication Type] OR random* [Title/Abstract] OR random allocation [MeSH Terms] OR therapeutic use [MeSH Subheading])	42	4	10	
12	(NTI-tss) AND ((clinical [Title/Abstract] AND trial [Title/Abstract]) OR clinical trials [MeSH Terms] OR clinical trial [Publication Type] OR random* [Title/Abstract] OR random allocation [MeSH Terms] OR therapeutic use [MeSH Subheading])	0			
13	(NTI*) AND ((clinical [Title/Abstract] AND trial [Title/Abstract]) OR clinical trials [MeSH Terms] OR clinical trial [Publication Type] OR random* [Title/Abstract] OR random allocation [MeSH Terms] OR therapeutic use [MeSH Subheading])	98	4	10	
14	(Nociceptive trigeminal inhibition) AND ((clinical [Title/Abstract] AND trial [Title/Abstract]) OR clinical trials [MeSH Terms] OR clinical trial [Publication Type] OR random* [Title/Abstract] OR random allocation [MeSH Terms] OR therapeutic use [MeSH Subheading])	25	4	10	
15	(Nociceptiv* trigeminal inhibition) AND ((clinical [Title/Abstract] AND trial [Title/Abstract]) OR clinical trials [MeSH Terms] OR clinical trial [Publication Type] OR random* [Title/Abstract] OR random allocation [MeSH Terms] OR therapeutic use [MeSH Subheading])	26	5	10	
16	(Anterior deprogrammer) AND ((clinical [Title/Abstract] AND trial [Title/Abstract]) OR clinical trials [MeSH Terms] OR clinical trial [Publication Type] OR random* [Title/Abstract] OR random allocation [MeSH Terms] OR therapeutic use [MeSH Subheading])	0			

Search strategy and results (search date: December 31, 2007)

**Table 3:** TRIP database.

**#**	**Search terms**	**Hits**	**Relevant hits**	**Cumulative relevant hits**	**(New) Relevant references**
1	NTI	evidence-based Synopses: 1	0		
		guidelines: 2	0		
		clinical questions: 1	0		
		E-textbooks: 1	0		
		other: 1	0		
		Medline:			
		therapy: 9	3	3	[9,10,22]
		diagnosis: 8	0		
		systematic reviews: 4	0		
		prognosis: 8	0		
		etiology: 5	0		
2	NTI-tss	0	0	3	
3	NTI*	systematic reviews: 207	0		
		evidence-based synopses: 17	0		
		guidelines: 33	0		
		clinical questions: 33	0		
		E-textbooks:19	0		
		More: 3	0		
		Medline:			
		therapy: 16	3	3	
		diagnosis: 11	0		
		systematic reviews: 26	0		
		prognosis: 16	0		
		etiology: 15			
4	nociceptive trigeminal inhibition	systematic reviews: 1	0		
		Guidelines: 3	0		
		E-textbooks: 9	0		
		Medline:			
		therapy: 6	4	6	[16-18]
		diagnosis: 2	0		
		systematic reviews: 0			
		prognosis: 1	0		
		etiology: 0			
5	nociceptiv* trigeminal inhibition	systematic reviews: 1	0		
		guidelines: 3	0		
		E-textbooks:9	0		
		Medline:			
		therapy: 8	5	6	
		diagnosis: 2	0		
		systematic reviews: 0	0		
		prognosis: 1	0		
		etiology: 0			
6	anterior deprogrammer	0			

Search strategy and results (search date: December 31, 2007)

**Table 4:** MedPilot.

**#**	**Search terms**	**Hits**	**Relevant hits**	**Cumulative relevant hits**	**(New) Relevant references**
1	NTI	All: 396			
		Medline: 357	7	7	[9,10,12,22,60-62]
		in others 5	0		
2	NTI-tss	0			
3	NTI*	825 Medline: 538	(see PubMed search)		
4	nociceptive trigeminal inhibition	157 Medline: 156	4	10	[17,16,18]
5	nociceptiv* trigeminal inhibition	157 Medline: 156	4	10	
6	anterior deprogrammer	Medline: 4	0	10	

Search strategy and results (search date: December 31, 2007)

**Table 5:** BIREME.

**#**	**Search terms**	**Hits**	**Relevant hits**	**Cumulative relevant hits**	**(New) Relevant references**
1	NTI	General Health Sciences (190):			
		LILACS: 5	0		
		Medline: 161	(see PubMed search)		
		Cochrane Library: 23	0		
		SciELO: 1	0		
		Specialized Areas (1):			
		BBO: 1	0		
		International Agencies (1):			
		WHOLIS: 1	0		
2	NTI-tss	0			
3	NTI*	0			
4	nociceptive trigeminal inhibition	General Health Sciences (114):			
		Medline: 102	(see PubMed search)		
		Cochrane Library: 12	0		
5	nociceptiv* trigeminal inhibition	0			
6	anterior deprogrammer	General Health Sciences (3):			
		LILACS: 1	0		
		Medline: 2	0		

Search strategy and results (search date: December 31, 2007)

**Table 6:** Database of the Deutsche Ärzte Verlag.

**#**	**Search terms**	**Hits**
1	NTI	0
2	NTI-tss	0
3	NTI*	0
4	nociceptive trigeminale Inhibition	0
5	nociceptiv* trigeminale Inhibition	0
6	anteriorer Deprogrammierer	0

Search strategy and results (search date: December 31, 2007)

**Table 7:** Database of the Deutsche Quintessenz Verlag.

**#**	**Search terms**	**Hits**
1	NTI	3062
2	NTI-tss	0
3	NTI*	0
4	nociceptive trigeminal inhibition	0
5	nociceptiv* trigeminal inhibition	0
6	anterior deprogrammer	0

Search strategy and results (search date: December 31, 2007)

**Table 8:** Google Scholar.

**#**	**Search terms**	**Hits**	**Relevant hits**	**Cumulative relevant hits**	**(New) Relevant references**
1	NTI	about 95,000			
2	NTI-tss	28	18	18	[1,3,8,10,16,24,26,36,63-72]
3	NTI*	93,400			
4	nociceptive trigeminal inhibition	8,760			
5	nociceptiv* trigeminal inhibition	18	2	20	[10,19]
6	anterior deprogrammer	37	2	20	

Search strategy and results (search date: December 31, 2007)

**Table 9:** Web of Science: Cited reference search.

**#**	**Search terms**	**Times cited**	**Relevant hits**	**Cumulative relevant hits**	**(New) Relevant references**
1	Baad-Hansen (2007)	0			
2	Aristeguieta (2006)	0			
3	Wright (2006)	0			
4	Jokstad (2005)	3	1	1	[22]
5	Magnusson (2004)	5	2	2	[10]
6	Shankland WE (2002)	N.N.			
7	Shankland WE (2001)	N.N.			

Search strategy and results (search date: December 31, 2007). N.N.: nomen nescio (unknown author)

The last update of all electronic searches was carried out on December 31, 2007.

In addition to the database search, textbooks related to the topics of TMDs, occlusion, and bruxism were considered. Furthermore, the Swiss provider (Karr Dental) and an international European provider of the NTI-tss device (e-motion) were requested to send us possible further material and publications. The abstracts, or, when available, the full-text papers were read in order to establish the acceptability of the publications to this review. Finally, the reference lists of the identified relevant articles were screened to find additional pertinent contributions (e.g., journal articles, textbooks, book chapters, master or doctoral theses, course material).

The strength of evidence related to the identified publications was evaluated using the classification suggested by Antes [14] (Table 10). Articles about randomized controlled trials (RCTs) were evaluated according to the quality score developed by Jadad et al [15]. The Jadad scale consists of five items, which focus on three dimensions of internal validity (randomization; double blinding; description of withdrawals and drop-outs) (Table 11). Since double-blinding (as required in the Jadad scale) is not possible when occlusal devices are used, single-blinding of the investigator(s) to the type of splint worn by the patient was used as criterion.

**Table 10:** Hierarchy of strength of evidence for therapeutic decisions [slightly modified after [14]]

**Level**	**Description**
I	Systematic review of randomized controlled trials (RCTs)
II	Article about an RCT
III	Article about an experimental study without randomization; cohort study; case-control-study
IV	Article about a non-experimental study (cross-sectional study; case series; case report)
V	Narrative review or expert opinion (based on clinical experience) without explicit clinical appraisal (e.g., statements, editorials; expert commentaries to published articles; interviews with experts, brief references to NTI-tss in articles; commercial-like reviews)

**Table 11:** Determination of the quality score proposed by Jadad et al [15]

Articles are assessed according to the following questions:
• Was the study described as randomized?
• Was the study described as double blind?
• Was there a description of withdrawals and dropouts? (The number *and *the reasons for withdrawal in each group must be stated. If there were no withdrawals, it should be stated in the article.)
*A score of 1 point is given for each "yes," a score of 0 points is given for each "no."*
• *One additional point *is given if for question 1 the method to generate the sequence of randomization was described *and *it was appropriate (i.e., if it allowed each study participant to have the same chance of receiving each intervention and the investigators could not predict which treatment was next).
• *One additional point *is given if for question 2 the method of double blinding was described *and *it was appropriate (i.e., if it is stated that neither the person doing the assessments nor the study participant could identify the intervention being assessed, or in the absence of such a statement the use of active placebos, identical placebos, or dummies is mentioned).
• *One point is deducted *if for question 1 the method to generate the sequence of randomization was described *and *it was inappropriate (e.g., patients were allocated alternately).
• *One point is deducted *if for question 2 the method of double-blinding was described *and *it was inappropriate.

Uncertainties on data interpretation and discrepancies in scoring according to the classifications by Antes [14] as well as Jadad et al [15] were resolved by discussion between the two reviewers.

### Identification of publications about complications and/or side effects of the NTI-tss device

For the identification of complications and/or side effects associated with the use of the NTI-tss device, pertinent reports found in the identified articles using the search strategies mentioned above were considered.

In addition, a specific search in the FDA's Manufacturer and User Facility Device Experience Database (MAUDE) was carried out with the following strategy: Go to Simple Search → Search term: NTI; Date Report Received by FDA: ALL YEARS

## Results

### Overall yield of the search

**Figure 3:**
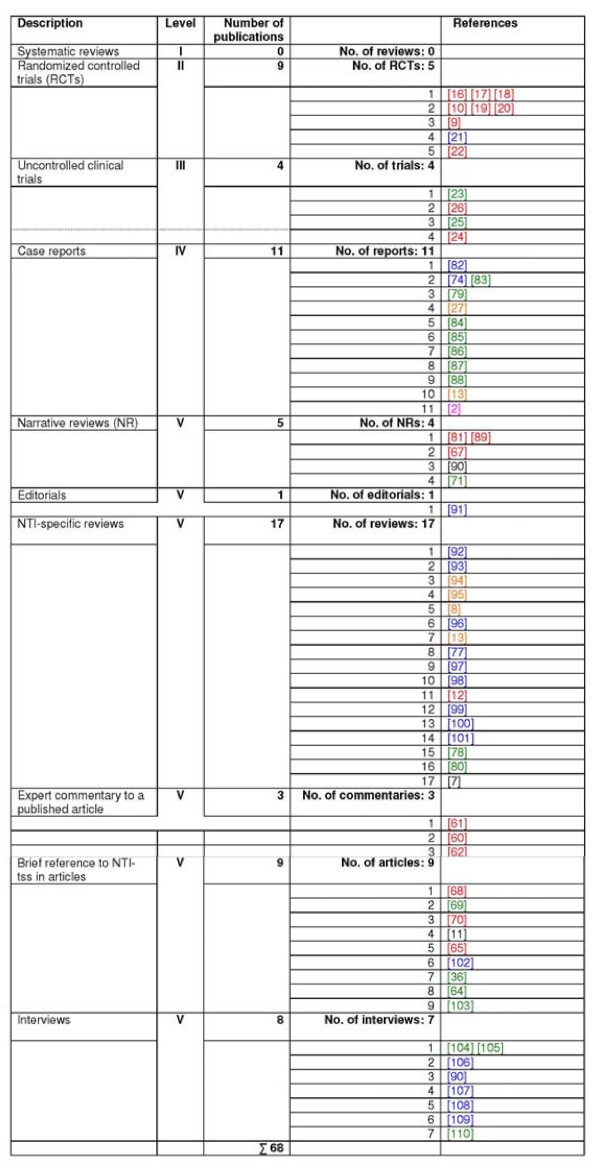
**Allocation of the identified 68 articles according to the hierarchy of strength of evidence**. Red: hits in electronic databases (n = 20); green: publications identified by searching the websites (n = 19); orange: articles found in the reference lists of identified articles (n = 6); purple: contributions found in textbooks (n = 1); blue: publications mailed by providers of the NTI-tss device (n = 19); black: publications found in other sources (n = 3).

A total of 68 relevant publications of different levels of evidence were identified. Details about the search results in the nine electronic databases as well as in the NTI-tss-related websites are shown in Tables 1 to 9 and 12, respectively. Figure 3 (right column) reveals by which search strategy the publications were found: only 39 contributions were identified by the search in electronic databases and websites.

**Table 12:** NTI-tss-related websites. Results

**Web link**	**Relevant hits**	**Cumulative relevant hits**	**Reference/Title**
	8	8	[10,16,22,23,26,48,73,74]
	3	11	[6,75,76]
	9	16	[25,77-80]
	4	18	[17,81]

### Qualitative analysis

**Figure 4:**
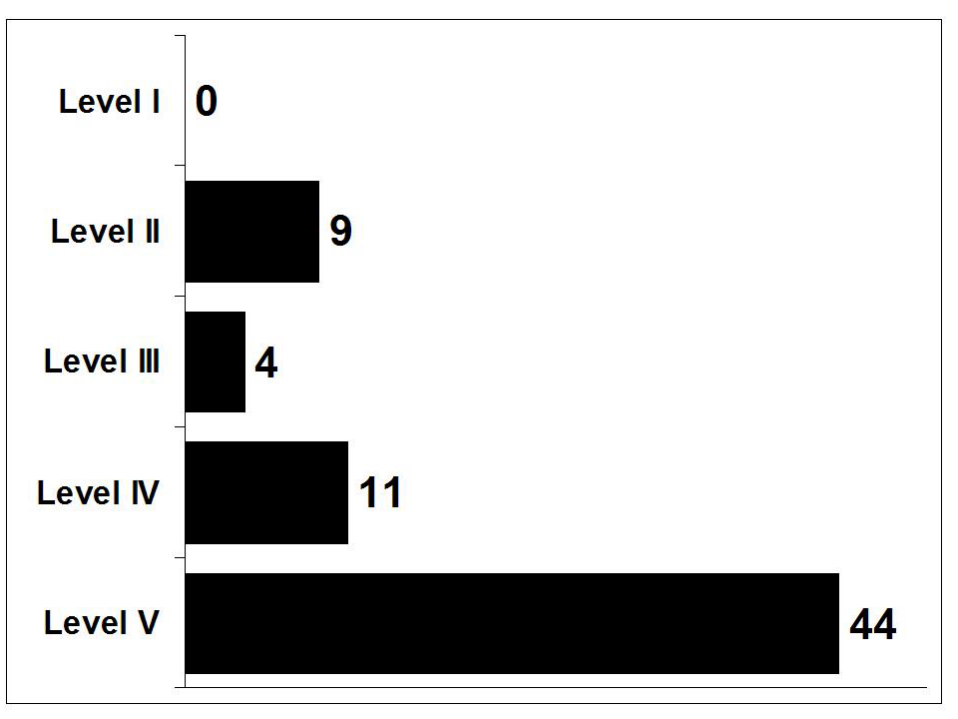
Qualitative distribution of the identified publications according to the strength of evidence (cf. Table 1).

The overall qualitative distribution of the relevant publications according to their strength of evidence is shown in Figure 4. Figure 3 provides detailed information about the allocation of the identified 68 articles into the different levels of evidence.

While no systematic review of RCTs (level I) could be identified, 13 contributions reported about the results of clinical studies. Nine of these publications [9,10,16-22] referred to 5 RCTs (level II). Seven of the nine publications about RCTs were published in dental journals; one was a congress abstract [19], and another one was a doctoral thesis [21]. Except the thesis, which was written in Turkish, and a Norwegian article [20], the papers were published in English. The allocation according to the Jadad scale is shown in Table 13.

**Table 13:** Assessment of the quality of the articles about randomized controlled trials (RCTs) according to the quality score proposed by Jadad et al [15]

**Study**	**Study described as randomized?**	**Method described *and *appropriate?**	**Study described as blind?**	**Method described *and *appropriate?**	**Description of withdrawals/dropouts?**	**Jadad score**
Baad-Hansen et al [22]	Yes	Not described	Yes	Yes	No withdrawals or dropouts	4
Kavaklı [21]	Yes	Not described	No	---	No withdrawals or dropouts	2
Jokstad et al [10,20]	Yes	Yes	Yes	Yes	Yes	5
Magnusson et al [9]	Yes	Yes	No	---	Yes	3
Shankland [16,18]	Yes	Not described	No	---	No	1

Among the four publications allotted to level III, there were three congress abstracts [23-25] and one article [26] about an uncontrolled clinical investigation. Twelve contributions referring to 11 case reports were categorized into level IV.

Forty-three publications were classified to level V, representing narrative reviews, editorials, NTI-specific reviews, brief references to NTI-tss in articles, expert commentaries, and interviews.

### Contents-based analysis of the articles on RCTs

The methods and results of the five RCTs are summarized in Tables 14 to 18. In four studies [9,10,21,22], a complete-arch, hard acrylic resin occlusal stabilization (i.e. non-repositioning) appliance, which was worn at night, was used as therapeutic comparison (Tables 14, 15, 16, 17). In the other trial [18], a full-coverage occlusal splint similar to a bleaching tray was chosen; it was used at night and during stressful periods during wakefulness. Two RCTs focused on electromyographic (EMG) investigations of jaw-closing muscles: In one trial, participants were diagnosed with TMDs and concomitant bruxism [22] (Jadad score: 4); in the other study, the only inclusion criterion was bruxism (and associated symptoms) [21] (Jadad score: 2). In both investigations, the NTI-tss device, but not the occlusal stabilization appliance, showed a significant reduction of the EMG activity. A decrease in clinical symptoms and signs (i.e., pain; number of muscles with tenderness upon palpation; maximum unassisted jaw opening) could not be observed [22].

**Table 14:** Characteristics of the study of Baad-Hansen et al [22]

**Study**	**Type of study**	**Aim of the study**	**Patient recruitment**	**Inclusion criteria**	**Exclusion criteria**	**n**	**Therapeutic comparison**
Baad-Hansen et al [22]	Investigator-blinded randomized controlled cross-over trial	1. To compare the therapeutic efficacy of two different intraoral devices on the EMG activity of bruxers during sleep.	Self-presentation of patients at the School of Dentistry, University of Aarhus (Denmark)	1. Self-reported tooth-grinding during sleep, confirmed by bed-partner.	Use of any other medication than mild analgesics	10 (average age: not reported; range: 23–39 years)	NTI-tss device (n = 10) vs. flat occlusal stabilization splint (OS) (n = 10) worn at night
				2. Reports of muscle soreness on awakening.			
				3. Signs of tooth wear.			
		2. To evaluate if changes in EMG activity are associated with short-term changes in TMD-related pain.		4. TMD diagnosis by a blinded investigator according to the RDC/TMD [111].			

**Study**	**Study duration**	**Outcome parameters**	**Results**			**Authors' conclusions**	

Baad-Hansen et al [22]	7–8 weeks	1. "A strong and lasting inhibition of EMG activity in masseter muscles during sleep was caused by wearing the NTI splint but not the OS. However, this was not directly related to the short-term clinical outcome measures."	**Within-treatment-group pre-post differences**			"A strong and lasting inhibition of EMG activity in masseter muscles during sleep was caused by wearing the NTI splint but not the OS. However, this was not directly related to the short-term clinical outcome measures	
			NTI-tss group:				
			- Significant decreases at all levels of EMG threshold during the use of the NTI-tss device when compared with baseline.				
			OS group:				
			- No differences between the baseline EMG values for any of the EMG measures.				
			**Between-groups post-treatment differences**				
			EMG outcomes:				
			In contrast to the OS, the NTI-tss device was associated with significant decreases when compared with EMG baseline values.				
			Clinical outcomes:				
			- No differences between the two therapies				
			- No significant correlations between EMG data and clinical variables.				

**Table 15:** Characteristics of the study of Kavaklı [21]

**Study**	**Type of study**	**Aim of the study**	**Patient recruitment**	**Inclusion criteria**	**Exclusion criteria**	**n**	**Therapeutic comparison**
Kavaklı [21]	Randomized controlled trial	To compare the therapeutic efficacy of two different intraoral devices on the EMG activity of bruxers during sleep.	Self-presentation at the Hacettepe University Health Science Institute, Ankara (Turkey)	1. Self-reported tooth clenching and tooth grinding for at least 6 month	1. More than two missing molars	20 (average age: 31 years; range: 14–52 years)	NTI-tss device (n = 11) vs. Michigan- type stabilization splint (SS) (n = 9) worn at night
				2. Grinding sounds during sleep for at least 3 nights per week as confirmed by bed-partner	2. Removable prosthetic restoration		
				3. Jaw muscle discomfort	3. Gross malocclusion		
				4. Abnormal tooth wear	4. Constant use of sleep medication		
				5. Masseter hypertrophy	5. Abuse of alcohol and/or drugs		
				6. Diagnosis of sleep bruxism in a sleep laboratory	6. Neurological or psychological diseases		
					7. Sleeping disorders		
					8. Internal TMJ derangements as diagnosed with an MRI		
	
**Study**	**Study duration**	**Outcome parameters**		**Results**		**Authors' conclusions**	
	
Kavaklı [21]	4 months	(A) Sleep variables:		**Within-treatment-group pre- post differences**		1. Both splint designs do not stop sleep bruxism activity as shown by polysomnographic evaluation.	
		- sleep quality					
		- total sleep time		NTI-tss device:		2. The SS does not reduce the frequency, duration or intensity of the sleep bruxism.	
		- sleep efficiency		- no changes of other sleep parameters		3. The NTI-tss device reduces the intensity of bruxism.	
		- sleep latency		- no changes of respiratory parameters		4. Due to its positive effect on sleep bruxism and its easy adapatability, the NTI-tss device is recommended if regular check-ups by a dentist are possible.	
		- REM latency		- no changes in occlusion			
		- percentages of stage duration		- reduced intensity of masseter and temporalis muscles contraction activities compared to baseline			
		- number of awakenings during sleep					
		- number of movements during sleep					
				Stabilization splint:			
		(B) Respiratory variables:		- sleep stage 2 was shorter as compared to baseline			
		- apnea					
		- hypoapnea		- no changes of other sleep parameters			
		(C) Bruxism-related variables as derived from masseter and temporal muscle activity:		- no changes of respiratory parameters			
		- total bruxism duration		- no changes of sleep bruxism activity			
		- number of bruxism episodes/night					
		- number of bruxism episodes/h		**Between-groups post- treatment differences** No changes			
		- number of bruxism burst/episode					
		- number of bruxism burst/h					
		- amplitude of bruxism episodes					

**Table 16:** Characteristics of the study of Jokstad et al [10,19,20]

**Study**	**Type of study**	**Aim of the study**	**Patient recruitment**	**Inclusion criteria**	**Exclusion criteria**	**n**	**Therapeutic comparison**
Jokstad et al [10,19,20]	Investigator-blinded randomized controlled trial	To compare the therapeutic efficacy of two different intraoral devices in TMD patients.	TMD patients referred to or applying for therapy at the Department of Prosthetic Dentistry and Stomatognathic Physiology, Faculty of Dentistry, University of Oslo (Norway)	Adults who had experienced TMD symptoms for at least 6 months, e.g.,	1. Partial protheses with distal extensions	38 (average age: 37 years; range: 17–62 years)	NTI-tss device (n = 18) vs. Michigan-type stabilization splint (SS) (n = 20)
				- impaired range of motion	2. Additional TMD therapy during the trial		
				- impaired TMJ function	3. Recent facial or cervical trauma		worn at night
				- muscle pain			
				- TMJ pain			
				- pain on mandibular movement			
							Plus (both groups):
							1. counseling
							2. muscle relaxation exercises
	
**Study**	**Study duration**	**Outcome parameters**		**Results**		**Authors' conclusions**	
	
Jokstad et al [10,19,20]	3 months	- Self-reported headache and TMD-related pain on a 100 mm VAS		**Within-treatment-group pre-post differences**		1. The therapeutic efficacy between an NTI-tss device and a Michigan splint did not differ over an observation period of three months.	
		- Maximum unassisted jaw opening		- Average jaw opening increased in both groups			
		- Tenderness on palpation of masticatory muscles, neck and shoulder muscles and TMJs on a 100 mm VAS		- VAS scores for TMD-related pain and self reported headache decreased significantly between baseline and 3-months examination in both groups			
				- Reduction of jaw muscle (but not neck and shoulder muscles) tenderness upon palpation in both groups			
				- No changes of TMJ tenderness upon palpation			
				**Between-groups post-treatment differences**			
		- Comfort of splint use on a 100 mm VAS		- No differences between NTI-tss and SS groups at the 3-months examination			
				- In both groups, the comfort of splint use appeared to be similar at each control examination with a (statistically not significant) trend of higher comfort for the NTI-device			

**Table 17:** Characteristics of the study of Magnusson et al [9]

**Study**	**Type of study**	**Aim of the study**	**Patient recruitment**	**Inclusion criteria**	**Exclusion criteria**	**n**	**Therapeutic comparison**
Magnusson et al [9]	Investigator-blinded randomized controlled trial	To compare the therapeutic efficacy of two different intraoral devices in TMD patients.	Patients referred for suspected TMDs to the Department of Stomatognathic Physiology, the Institute for Postgraduate Dental Education, Jönköping (Sweden)	1. TMD symptoms ≥ 6 months	1. Ongoing TMD therapy	28 (average age: 31.5 years; range: 16–70 years)	NTI-tss device (n = 14) vs. Michigan-type stabilization splint (SS) (n = 14) worn at night
				2. Age: ≥ 16 years	2. Therapy with any kind of interocclusal appliance during the past year		
				3. ≥ 12 teeth both in the upper and lower jaw	3. TMD symptoms and signs justifiying another initial therapy than an interocclusal appliance		
				4. Moderate or severe clinical signs according to Helkimo's Clinical Dysfunction Index	4. Anterior teeth with periodontitis or fixed partial dentures		
				5. Pronounced subjective symptoms according to Helkimo's Anamnestic Index	5. Pronounced pre- or postnormal occlusion		
				6. Frequent tension-type headache	6. Deep bite		
					7. Psychic disorder		
					8. Language difficulties		
	
**Study**	**Study Duration**	**Outcome parameters**		**Results**		**Authors' conclusions**	
	
Magnusson et al [9]	6 months	- Mandibular mobility		**Within-treatment-group pre-post improvement**		1. "The almost 100% treatment success that have been claimed after NTI treatment could not be confirmed in this study."	
		- Deviation/deflectio		6-month follow-up of the NTI-group (n = 10):		2. "The statement of a gain in chair-side time with the NTI device can be questioned. The use of NTI devices, however, eliminates one clinical visit, as well as the laboratory costs."	
		- n on jaw		- significant improvement (n = 6)		3. "It can not be ruled out that long time use of NTI splints can be detrimental for the occlusion."	
		- opening		- some improvement (n = 1)		"All of the studied variables were in favour for the stabilization splint, compared to the NTI device."	
				- no change (n = 2)			
		- TMJ sounds		- impairment (n = 1)			
		- Pain on mandibular movement		- impaired occlusion (n = 1)			
		- Muscle/TMJ pain upon palpation		- decreased use of analgetics (n = 2)			
		- Number of occluding teeth		- increased use of analgetics (n = 2)			
		- Time for impression-taking, interocclusal recording and adjustment of stabilization splint		- 6-point behavior scale: 3.4 → 2.1			
		- Time for fitting and adjustment of the NTI-tss device		- 11-point numerical scale: 5.9 → 3.8			
		- Adherence to the allocated splint		6-month follow-up of the SS-group (n = 14):			
		- Use of analgetics		- significant improvement (n = 12)			
		- Symptom intensity (6-point behavior scale, 11-point numerical scale)		- some improvement (n = 2)			
				- decreased use of analgetics (n = 10)			
				- 6-point behavior scale: 3.4 → 1.9			
				- 11-point numerical scale: 5.9 → 3.3			
				**Between-groups post-treatment differences**			
				- 3-month follow-up: 4 patients with NTI-tss device changed to SS due to impairment or no improvement of their symptoms, while none of the SS users changed the group.			
				- For all variables, improvement was larger in the SS-group than the NTI-group			
				- SS-appliances were judged to be more comfortable than the NTI-tss device			
				**Mean production time**			
				- NTI-tss device: 27 minutes			
				- SS: 17 minutes			

**Table 18:** Characteristics of the study of Shankland [16,18]

**Study**	**Type of study**	**Aim of the study**	**Patient recruitment**	**Inclusion criteria**	**Exclusion criteria**	**n**	**Therapeutic comparison**
Shankland [16,18]	Randomized controlled clinical trial	To evaluate the safety and effectiveness of the NTI-tss device for the reduction of frequency and severity of tension-type and migraine headaches.	Not reported	1. Diagnosis of tension-type or migraine headaches	Presence of TMDs	94 (average age and range: not reported)	NTI-tss device (n = 51) vs. full-coverage occlusal splint without cuspid rise and anterior guidance (similar to a bleaching tray) (n = 43) worn at night and during the day when perceiving stressful periodes
				2. Intake of Sumatriptan as a rescue medication			
				3. 3. Having experienced an average of two migraine episodes or 8 tension-type headaches per month			
				4. Presence of natural or fixed prosthetic maxillary and natural mandibular incisors			
				5. Overbite and overjet within normal limits			
				6. Stable dentition with no current orthodontic treatment and fully erupted teeth			
				7. No significant periodontal disease			
				8. No TMD signs and/or symptoms			
				9. At least 18 years of age			
	
**Study**	**Study duration**	**Outcome parameters**		**Results**		**Authors' conclusions**	
	
Shankland [16,18]	4 weeks baeline data + 8 weeks therapy	(A) Clinical objective outcomes:		**Within-treatment-group pre-post differences**		1. "The NTI-tss appliance proved safe and efficacious in the reduction of medically diagnosed migraine and tension-type headache."	
		- Interocclusal record		NTI-tss group:			
		- Tooth mobility of all anterior teeth		- 16% of the participants reported an 85% to 100% reduction of migraine headaches			
		- Tooth sensibility of the anterior teeth (temperature, pressure)		Users reported an elimination of 46.9% of all headache pain as well as reduction of photophobia, phonophobia and nausea			
		- Periodontal health of all teeth		Control group:			
		- Periapical radiographs of the anterior teeth		- 7% of the subjects reported a 85% to 100% reduction of migraine headaches			
		- Vitality of the anterior teeth		- 27% of the subjects reported an average 46% increase in migraine events			
		- Tenderness of the head and neck muscles		- Increase of migraine frequency			
		- Trigger points		**Between-groups post-treatment differences**			
		- Range of mandibular motion		- In every category (headache episodes, dosages of rescue medicine (Imitrex^®^), phonophobia, photophobia, nausea), the percentage of reduction of all symptoms decreased for those in the NTI-tss group.			
		- TMJ noises					
		- TMJ tenderness					
		- Tension-type headache					
		- Migraine headache					
		- Nausea					
		- Photophobia					
		- Phonophobia					
		- Analgesics taken (and doses)					
		- Use of rescue medicine (Sumatriptan^®^)					
		(B) Clinical subjective outcomes (VAS):					
		- Intensity of tension-type headache					
		- Intensity of migraine					

Two RCTs investigated clinical variables in TMD patients. One of these trials [10] (Jadad score: 5) found no difference between these two devices, while in the other study [9] (Jadad score: 3) a stabilization appliance led to a greater improvement than an NTI-tss device. However, only descriptive statistics were provided in the latter study report. In one RCT [18] (Jadad score: 1), patients with migraine or tension-type headache responded more favorably to the therapy with an NTI-tss device than with a bleaching tray-like splint.

### Complications/side effects related to the NTI-tss-device

While no incidents occurred in the RCTs of Baad-Hansen et al [22] and Shankland [16-18], complications or side effects were observed in the other three RCTs [22] (Table 19). Two more incidents [13,27] were identified in the reference lists of Magnusson et al [9] and Jokstad et al [10], respectively, while one report was found in a book chapter [2]. Finally, five cases were published in the FDA Medical Device reporting website (Table 20).

**Table 19:** Complications and side effects associated with the use of the NTI-tss device as described in the identified publications

**Study**	**Complications/Side effects**
Clark & Minakuchi [2]	• Anterior open bite induced after wearing the device 24 hours a day over an extended period of time (n = 1)
Kavaklı [21]	• Radiologically diagnosed widened periodontal ligament plus alveolar bone resorption in one tooth (n = 1/20)
Jokstad et al [10]	• Sensitive lower front teeth (n = 1/18)
	• Dryness of the mouth while sleeping (n = 6/18) due to a forced open jaw caused by the NTI-tss design
	• Swallowing difficulties (n = 2/18)
	• The protruding tip of the device was felt to be annoying (n = 1/18)
	• The device was falling out or being taken out unconsciously while sleeping (n = 2/18)
Magnusson et al. [9]	• Altered occlusion between the 3- and 6-month follow-up: "the vertical overbite decreased with one millimeter, and the number of occluding tooth pairs was reduced from 10 to 15" (n = 1/14)
	• Transient tenderness in the teeth when the device was used (n = 6/14)
	• Tongue thrusting, dry mouth, increased salivation, snoring (as reported by "single patients")
Fleten & Gjerdet [13]	• Swallowing of an NTI-tss device, which was lodged at the esophageal entrance (n = 1)
Fitins [27]	• Sensitive and painful maxillary central incisor (n = 1)
	• Moderate mobility of and local bone loss around three maxillary incisors

**Table 20:** Complications and/or side effects as described in the MAUDE database

**Web link**	**Year**	**Complaints/Side effects**
	2003	"This device [...] will not stop migraines, it took out pt's two front crown teeth, it causes tooth movement, joint strain [...]. This device is harmful – dangerous and it is not therapeutic. Pt had to have surgery to undo the harm that was caused by the nti splint"
	2004	"[...] the device was not only ineffective, but has done damage to their jaw alignment, bite, and facial appearance. The device caused them to grind their front teeth instead of their molars. Even though rptr only wore it for two weeks, rptr's jaw and their bite has not returned to "normal". During the two weeks rptr wore it, rptr suffered extreme headaches, facial pain, and their front teeth loosened. [...] Rptr's teeth are actually moving position since rptr can barely bite down without forcing their lower jaw back. Their front teeth are shifting back because they are constantly pushing against their upper teeth. Eating and chewing is challenging since they can't actually touch their upper and lower molars together without effort. Therefore, rptr's lower jaw spasms when they chew."
	2004	"Pt's concern is that this device poses a significant threat of death by choking. [...] A user could dislodge the nti-tss with the tongue, or, during sleep, unconsciously reach into the mouth to loosen or readjust it it is very uncomfortable-. If it remained in the mouth it could easily become lodged in the throat."
	2006	"Since the implant was put in, patient has been having migraine headaches and a clicking jaw. [...] Her teeth have moved so much that according to the dentist, she would need braces to move her teeth to position. Patient never had a history of migraine."
	2006	"Not only is his new apparatus devoid of a safety device to keep him from swallowing it in his sleep, but (1) it fit very tightly and (2) it was very difficult to remove. When he did take out the device, it felt as if he was going to pull his teeth out. Last night, when he went to sleep, he was awakened when the device broke in his mouth."

Different complications and side effects were documented; however, no case of an aspiration could be found.

## Discussion

We were surprised to find that within the past decade as much as nine different trials related to the NTI-tss device were carried out, five of which were RCTs, while four were uncontrolled investigations [23-26] (Figure 1). As far as the estimation of the efficacy of a therapeutic measure is concerned, it is wise to consider primarily (or exclusively [28]) articles about RCTs, because they provide the strongest evidence on the efficacy of a therapy [29,30]. Numerous examples have shown that observational studies, as compared to RCTs, are likely to generate over-optimistic (i.e., false-positive) conclusions [31-33]. Hence, it is not surprising that in clinical trials without control group the NTI-tss device was reported to be associated with a marked decrease in patients suffering from headache [25] and TMD-associated otologic symptoms (vertigo; otalgia; otic fullness; tinnitus; subjective hearing loss) [26].

In the five RCTs, which evaluated clinical and electromyographic effects of the NTI-tss device, different inclusion criteria and methods were used. Therefore, the results had to be analyzed separately. Each RCT has methodological weaknesses. Four studies had a limited number of participants (n = 10 to 38) (cf. Tables 14, 15, 16, 17, 18), which may have resulted in a statistical type II error (erroneous acceptance of the null hypothesis), as noted by Jokstad et al [10]. Shankland's investigation, in contrast, included 94 patients [18]; however, study and reporting quality were limited. The methodological flaws inherent to this study are not only reflected by the low Jadad score of 1, but also by the shortcoming to distinguish between the diagnoses of migraine and tension-type headache, which are completely different entities [34]. Shankland excluded patients with TMDs; however, there is a considerable symptom overlap between headache and TMD patients [35]. In fact, the same patient who suffers from pain in the temples may be diagnosed as having tension-type headache by a neurologist, whereas she may be diagnosed with myofascial pain in the temporal muscles by a dentist. Hence, an exact differential diagnosis between the two entities appears to be nearly impossible. Furthermore, there are limitations associated with the statistical analysis of the data gained in this study; for example, no information was provided about pre-treatment days of headache in that study [36].

Focusing on the identified articles about RCTs, two main indications for the NTI-tss splint may be distinguished: bruxism and TMDs. As far as bruxism is concerned, the studies of Baad-Hansen et al [22] and Kavaklı [21] have shown that – when compared to baseline EMG recordings from the masseter muscle during sleep – insertion of the NTI-tss device leads to a significant reduction in EMG activity of jaw closing muscles during clenching or grinding. These findings are compatible with early results from Van Eijden et al [37] who found that maximal effort clenches on the incisal edges of the incisors resulted in a significant decline of EMG activity as compared to clenching in intercuspation.

The fact that Baad-Hansen et al [22] were unable to correlate their EMG findings with clinical symptoms (e.g., reduction of reported pain) may be due to the short duration of wearing the intraoral device (2 weeks). Data from other investigations suggests that a decrease of EMG activity may indeed be associated with a pain reduction in patients with masticatory muscle pain [38]. Moreover, a decrease of EMG activity is not a unique characteristic of the NTI-tss device, but may be achieved with any anterior bite stop [39], sometimes even with a full-coverage occlusal appliance [38]. Hence, we concur with Clark and Minakuchi [2] who suggest in their recent review on oral appliances that it is "reasonable to use a partial-coverage anterior bite appliance in those patients with a known tooth-clenching habit, because this habit cannot be controlled with a stabilization appliance." On the other hand, a hard acrylic resin stabilization splint may be helpful in patients with tooth grinding, because it helps preventing the unwanted consequence of sleep bruxism [40], such as tooth wear (attrition), tooth grinding sounds, and – often – associated pain [41].

Regarding the management of patients with (localized) TMDs (e.g., myofascial face pain and/or arthralgia of the TMJs), the use of stabilization appliances is sufficiently supported by evidence in the dental literature [42-45]. Türp et al [45], for instance, concluded after a systematic review of the literature that most patients with masticatory muscle pain are helped by the incorporation of a stabilization splint. Particularly patients with local pain of the myofascial muscles (as opposed to individuals with widespread pain) are likely to experience improvement from this therapy [46].

Helkimo's statement that there is "no peer-reviewed scientific publication that exhibits that the NTI-splint is superior to other well-established conventional splints for the treatment if *[sic] *functionally related facial pain or mandibular dysfunction" [8] still holds true: a superiority of this anterior bite stop has not yet been shown. Nonetheless, the currently best available evidence [10] suggests that for the management of TMDs the NTI-tss device is similarly efficacious as a stabilization appliance. This demonstrates, on the other hand, that the observed reduction in clinical symptoms is not a feature unique to the NTI-tss device. Instead, it may also be achieved with other splints, even with other forms of anterior bite stops: Nilner et al [47], for instance, observed in patients suffering from myofascial face pain that at 6- and 10-weeks follow-up appointments a prefabricated appliance covering the six upper front teeth may be as effective in pain improvement as a traditional stabilization appliance. In a patient group with the same diagnosis, Al Quran and Kamal [48] who inserted an NTI-tss-like device (AMPS: anterior midline point stop) reported similar clinical results after 1 month and 3 months.

The positive effect after the incorporation of any type of appliance has frequently been explained by patient-specific behavioral changes [2]. However, such an assumption is hard to justify given the fact that the splints are usually worn at night during sleep, as it was also the case in the RCTs reviewed here. Regarding localized myofascial pain of the masticatory musculature, an alternative explanation is based on the heterogeneous activation capability of these muscles [37,49-51]: Experimental results in healthy volunteers indicate that a temporary positional alteration of the mandible (e.g., an increase of the vertical dimension after insertion of an occlusal appliance) changes the intramuscular recruitment pattern, which is often followed by a pain reduction [52-55]. Although this biomechanical hypothesis needs further studies to be validated [56], it provides a physiologically plausible explanation for the therapeutic success gained with oral splints.

We were unable to find evidence for the statement that the NTI-tss device was indicated for the prevention of bruxism, TMDs, chronic tension-type headaches, migraine, or occlusal trauma. Likewise, due to the poor quality of the publications by Shankland [16,18,17], its use for the therapy of migraine remains doubtful.

Contrary to claims made in the dental literature [10,12], cases of aspirated NTI-tss devices could not be identified in the present review (c.f. Tables 19 and 20). None of the five reports published in the MAUDE database referred to such an event. Fleten and Gjerdet [13], who allegedly reported about an aspiration, presented a case where an NTI-tss splint was lodged at the esophageal entrance.

Nonetheless, possible side effects, particularly those related to the teeth and the occlusion, remain the greatest concern when wearing NTI-tss splints (cf. Table 20). Of course, adverse events may also occur when a stabilization appliance is used. Clark and Minakuchi [2] mention two concerns which, albeit rare, need to be considered in this context: (a) increase of clenching activity when wearing the bite splint; (b) unintended occlusal change after full-time use, due to alterations of the position of individual teeth or of the mandible. However, if a stabilization appliance is worn at night only – and, if needed (e.g., during stressful periods of life), additionally during one or two hours of the day – the development of an appliance-induced malocclusion is unlikely. Other side effects, such as increased or decreased salivation [57], are usually short-lasting, while tension in the teeth [57] can mostly be eliminated by careful adjustments along the labial and/or buccal surfaces that ensure the retention of the stabilization appliance. Since it is certain that published case reports represent only a fraction of all adverse events that have occurred while using any type of intraoral appliance, clinicians should be encouraged to frankly report any unintended negative outcome associated with splint therapy. In the hierarchy of scientific evidence, case reports represent a low level (cf. Table 9). Nonetheless, considering the absence of other data we believe that the publications summarized in Tables 19 and 20 deliver valuable information and convey an important message to the clinician (as well as to the patient), namely that the use of the device is not fee of risks. Unfortunately, no risk quantification is possible at this point in time. Meanwhile, it remains crucial to take care that a patient receiving an NTI-tss device remains compliant with follow-up appointments, especially when wearing it over an extended period of time.

It has been argued that an advantage of the NTI-tss splint as compared to a conventional occlusal bite splint is the reduced chair-side time. Therefore, some dentists are likely to be surprised when confronted with Magnusson et al's report [9] that on the average 27 minutes (range: 17–45 minutes) were needed to fit and adjust an NTI-tss device as opposed to 17 minutes (range: 11–26 minutes) for making interocclusal records, taking impressions, and adjusting the surface of a stabilization appliance. Indeed, this computation appears to be debatable. In a previous study it was shown that the average time required for chair-side adjustment of a stabilization appliances (fabricated without and with face-bow) was less than 11 minutes (range: 4–27 minutes) [58]. To determine centric relation by fabricating an interocclusal record and to make alginate impressions of the upper and lower jaw one may calculate additional 15 minutes. Hence, for making a stabilization appliance the dentist needs to be at the chair for about 25 to 30 minutes, which corresponds to the time frame reported by Magnusson et al [9] for preparing an NTI-tss device. Yet, it should be noted that an inexperienced clinician may need considerably more time for either device.

The occlusal stabilization splint [59] remains the "gold standard" for the therapy of patients with (particularly localized) temporomandibular pain (i.e., myofascial pain of the masticatory muscles; TMJ arthralgia) and/or bruxism because it "is an easily used, potentially long-term, and clinically effective treatment intervention with reasonable nightly patient compliance and good outcomes," thereby exhibiting "few potential complications [2]."

## Conclusion

There is evidence from RCTs that the NTI-tss bite stop may be successfully used for the management of TMDs and bruxism. However, to avoid potential side-effects, it must be ensured that the patient is willing to return regularly to the dentist's office for control sessions and, if needed, re-adjustments. If this prerequisite is fulfilled, the NTI-tss splint may be particularly justified in the following clinical situations:

• A patient with acute and intense temporomandibular pain (possibly accompanied by a restricted jaw opening), who requires (as an emergency therapy) the rapid incorporation of an oral appliance to increase the vertical dimension of the jaws.

• A reduction of the EMG activity of jaw closing muscles during jaw clenching or tooth grinding is desired.

## Competing interests

The authors declare that they have no competing interests.

## Authors' contributions

JCT designed the systematic review, HS carried out the systematic literature search. HS and JCT appraised the identified publications, and drafted the manuscript. Both authors read and approved the final manuscript.

## Pre-publication history

The pre-publication history for this paper can be accessed here:


